# Increased urinary IgM excretion in patients with chest pain due to coronary artery disease

**DOI:** 10.1186/1471-2261-13-72

**Published:** 2013-09-13

**Authors:** Rafid Tofik, Ulf Ekelund, Ole Torffvit, Per Swärd, Bengt Rippe, Omran Bakoush

**Affiliations:** 1 Department of Emergency Medicine, Lund University, Lund, Sweden; 2 Department of Nephrology, Lund University, Lund, Sweden; 3 Department of Internal Medicine, UAE University, Al Ain, UAE

**Keywords:** Urine IgM, Microalbuminuria, Acute coronary syndrome, Chest pain, Cardiovascular mortality

## Abstract

**Background:**

Micro-albuminuria is a recognized predictor of cardiovascular morbidity and mortality in patients with coronary artery disease. We have previously reported, in diabetic and non-diabetic patients, that an increased urinary excretion of IgM is associated with higher cardiovascular mortality. The purpose of this study was to investigate the pattern of urinary IgM excretion in patients with acute coronary syndrome (ACS) and its correlation to cardiovascular outcome.

**Methods:**

Urine albumin, and IgM to creatinine concentration ratios were determined in 178 consecutive patients presenting with chest pain to the Department of Emergency Medicine (ED) at the University Hospital of Lund. Fifty eight (23 female) patients had ACS, 55 (19 female) patients had stable angina (SA), and 65 (35 female) patients were diagnosed as non-specific chest pain (NS).

**Results:**

Urine albumin and IgM excretions were significantly higher in patients with ACS (p = 0.001, and p = 0.029, respectively) compared to patients with NS-chest pain. During the 2 years follow-up time, 40 (19 female) patients suffered a new major cardiovascular event (ACS, acute heart failure, stroke) and 5 (4 male/1 female) patients died of cardiovascular cause. A high degree of albuminuria and IgM-uria significantly predicted cardiovascular mortality and morbidity (HR = 2.89, 95% CI: 1.48 - 5.66, p = 0.002). Microalbuminuric patients (≥3 *mg*/*mmol*) with high IgM-uria (≥0.005 *mg*/*mmol*) had a 3-fold higher risk for cardiovascular new events compared to patients with low IgM-uria (RR = 3.3, 95% CI: 1.1 - 9.9, p = 0.001).

**Conclusion:**

In patients with chest pain, an increased urine IgM excretion, is associated with coronary artery disease and long-term cardiovascular complications. Measuring urine IgM concentration could have a clinical value in risk stratification of patients with ACS.

## Background

The number of patients presenting to the emergency department (ED) with symptoms consistent with acute coronary syndrome (ACS) is rising [1,2]. Medical history and ECG findings are widely used and reliable keystones in risk stratification of patients presenting with chest pain [3,4]. However, many patients who are admitted to the medical ward due to chest pain will not turn out to suffer from acute coronary syndrome (ACS) [5]. Thus better tools for risk stratification are needed.

ACS is associated with increased urinary albumin excretion [6,7]. Microalbuminuria (MA) has been recognized as an independent prognostic marker for poor outcome in patients with coronary artery disease (CAD) [8]. MA could reflect widespread endothelial dysfunction due to atherosclerotic disease [9], with relative lack of endothelial nitric oxide (NO) production and increased levels of oxidative stress [10]. Long-term follow up studies on proteinuric patients have revealed that IgM-uria may be associated with a higher risk for cardiovascular (CV) mortality [11-13]. For instance, type 2 diabetic patients with MA and IgM-uria had a 3-fold higher risk for CV mortality than patients with merely MA. To the best of our knowledge, IgM-uria has not been studied in patients with CAD. Therefore, in this cohort of patients presenting with chest pain to the emergency department (ED) we studied the diagnostic and prognostic value of IgM-uria.

## Methods

Consecutive patients presenting with symptoms suggestive of ACS, to the ED at Skåne University Hospital in Lund during 53 daytime working shifts between September 1^st^ and December 31^st^ 2010, were invited to participate in the study. The study was carried out in compliance with the Helsinki Declaration, and was approved by the Regional Ethical Review Board of Lund (Dnr 2009/441).

After informed consent, 178 (77 female) consecutive patients with chest pain were included in the study. The patients were further assessed by the attending physician. The physician assessment included history, physical examination, 12-lead electrocardiography (ECG) and cardiac Troponin T (TNT) assay. Vital signs were measured in supine position at admission to the ED. Mean arterial blood pressure (MAP) was calculated by adding one third of the pulse pressure to the diastolic blood pressure. All participants were asked at the time of admission to provide urine samples for urine protein analysis.

65 patients (35 female) with normal 12-lead ECGs and normal plasma TNT levels were discharged to go home after a few hours of observation in the ED (Table 1). 113 (46 female) patients were admitted to the medical ward with suspected acute coronary syndrome (ACS). Cardiac stress test (including exercise test or myocardial scintigraphy) was done in ambiguous cases (n = 36).

**Table 1:** Baseline characteristics and two-year cardiovascular outcome of 178 patients presented with chest pain to the emergency department divided into 3 groups according to the clinical diagnosis of coronary artery disease

	**Acute coronary syndrome**	**Stable Angina**	**Non-specific chest pain**	***P value *(ACS *vs. *NS)**
	**(ACS)**	**(SA)**	**(NS)**	
	***n =*** **58**	***n =*** **55**	***n =*** **65**	
Age, *yr*	76 (52-98)	70 (39-91)	62 (40-92)	*<0.001*
Sex, *n*	35/23	36/19	30/35	*0.1*
P. creatinine, *μmol/L*	86.5 (56-249)	85 (49-259)	75 (49-166)	*<0.001*
MAP, *mmHg*	106.7 (77-139)	105 (81-140)	105 (82-140)	*0.7*
BMI	25 (16.7-38.8)	27.6 (20.8-43.1)	27.2 (20.9-42.7)	*0.043*
Smoking,%	27.6	16.4	26.2	*0.1*
Hypertension,%	67.2	81.8	27.7	*<0.001*
Family history,%	41.4	43.6	32	*0.1*
DM (type 1/type 2)	10 (2/8)	18 (0/18)	6 (0/6)	*0.2*
LDL cholesterol, mmol/L	3 (1.3-5.4)	2.2 (1-5)	3.2 (1.8-5)	*0.5*
TGL, mmol/L	1.25 (0.6-6.6)	1.25 (0.6-3.5)	1.55 (0.7-4)	*0.2*
P. IgM, *g/l*	0.76 (0.2-3.5)	0.73 (0.05-1.5)	0.71 (0.27-4.9)	*0.9*
IgM-uria, *mg/mmol*	0.0066	0.0058	0.0046	*0.029*
	(0.00027-0.249)	(0.00075-0.0549)	(0.00098-0.0716)	
Albuminuria, *mg/mmol*	2 (0.1-247)	0.6 (0.1-59)	0.1 (0.1-70)	*0.001*
IgG-uria, *mg/mmol*	2.1 (0.1-89)	1.6 (0.1-6.6)	1.6 (0.1-11)	*0.06*
CRP, *mg/l*	3.1 (0.6-80)	1.4 (0.6-24)	1.6 (0.6-33)	*0.012*
TNT, *ng/l*	24 (5-1168)	9 (5-419)	5 (5-44)	*<0.001*
Admitted, *n (m/f)*	53 (34/19)	28 (16/12)	32 (17/15)	*<0.001*
CV outcome at 2 years follow up, *n (m/f)*	27 (16/11)	12 (6/6)	6 (3/3)	*<0.001*
	Death = 3 (2/1)	Death = 2(2/0)	Death = 0	
	AMI = 8(5/3)	AMI = 3(1/2)	AMI = 3(2/1)	
	Angina = 9(5/4)	Angina = 3(1/2)	Angina = 1 (0/1)	
	CVI = 1 (0/1)	CVI = 0	CVI = 1(1/0)	
	HF = 6 (4/2)	HF = 4(2/2)	HF = 1 (0/1)	

**MAP**: mean arterial pressure, **BMI**: body mass index, **Admitted**: number of patients admitted to hospital. **DM:** Diabetes mellitus, **IgM-uria:** Urine IgM creatinine ratio, **CRP:** C-reactive protein, **TNT:** Troponin T. **LDL cholesterol:** low density lipoprotein cholesterol level, **TGL:** triglycerides level.

The final diagnosis was retrieved from the discharge notes of the patient’s medical records. Patients who were diagnosed with ACS were those with acute ST-elevation (STEMI) or non ST-elevation myocardial infarction (NSTEMI) or unstable angina. Patients with a previous history of cardiovascular event (angina, acute myocardial infarction (AMI), stroke), with no emerging ECG changes and normal TNT levels were diagnosed as stable angina (SA). Patients with atypical anginal chest pain with no history of previous cardiovascular event with normal ECG and TNT series were diagnosed as non-specific chest pain (NS).

The patients were followed up to the end of February 2012 for occurrence of any major cardiovascular event such as death, MI, and stroke. Cardiovascular death was defined as all deaths where unequivocal non-CV death was not established.

### Laboratory methods

The plasma and urine creatinine (enzymatic method), serum and urine proteins, and serum lipids were analyzed at the Central Clinical Chemistry Laboratory at the University Hospital in Lund, for details see [14,15]. Patients were defined as having dyslipidemia if serum low density lipoprotein cholesterol >3 mmol/L and/or serum triglycerides level >1.7 mmol/L [16]. Urinary albumin concentration was measured by immunoturbidometry on fresh urine samples taken at the time of patient admission. We used a Cobas Mira S system (Roche Inc.) and monospecific rabbit antisera obtained from Dako (Copenhagen, Denmark). Urine samples were stored in 3.5-ml polypropene tubes and kept at –20C until analysis for IgM. Urine IgM concentrations were measured by an ELISA technique described in detail elsewhere [17]. The lower detection limit for the urine IgM assay is 1 μg/l; the intra-assay and inter-assay variation is 4.6% and 10.9%, respectively.

Urine albumin, and IgM are presented as albumin, and IgM-creatinine ratios mg/mmol (ACR and MCR), respectively. The lowest detection level of ACR is 0.1 mg/mmol. The median value of urine IgM concentration of the whole cohort was 0.005 mg/mmol. Patients were divided into low and high IgM-uria groups according to the cohort median concentration.

### Statistical methods

Data are presented as medians (and ranges). Statistically significant levels were set considering 95% confidence intervals (CI) and a P value <0.05. Kruskal–Wallis H and Mann-Whitney U tests were used to compare groups. Kaplan–Meier curves (for survival analysis) and Log Rank test were used to assess differences in survival. Cox-proportional hazards regression analysis was employed to assess the association of baseline variables and cardiovascular outcome. Patients with missing data were excluded. All statistical analyses were performed by using IBM SPSS statistics for Windows, version 17.0 (SPSS).

## Results

Fifty eight (23 female) patients had acute coronary syndrome. 3 (1 female) patients with STEMI, 25 (8 female) with NSTEMI and 30 (14 female) patients had unstable angina. Fifty five (19 female) patients had SA. Sixty five (35 female) patients were diagnosed as NS.

There was no difference in gender, mean arterial blood pressure, smoking, and family history of IHD between patients with ACS and NS chest pain (Table 1). Patients with ACS were older with higher prevalence of hypertension, worse kidney function and higher hs-CRP than NS patients. Urine albumin and urine IgM excretions were higher in patients with ACS than those with NS (*p =* 0.001 and *p =* 0.029, respectively), as shown in Table 1. However, there was no difference in urine IgM or urine albumin excretion between patients with SA and NS (*P = 0.3, P = 0.2, ns*) or between patients who performed the stress test or not (*P = 0.3, P = 0.5, ns*).

During a 2 year follow-up, 5 (1 female) patients died from a cardiovascular cause and 40 (19 female) patients suffered a new major cardiovascular event, (Table 1).

### Albumiuria and IgM-uria in risk stratification of patients with chest pain

By univariate Cox-regression analysis the predictors for major cardiovascular events were: age (HR = 1.031), hypertension (HR = 2.38), previous ischemic heart disease (HR = 4.33) hs-CRP (HR = 1.87), TNT (HR = 2.02), albuminuria (HR = 1.87), and IgM-uria (HR = 2.96), Table 2. Diabetes, dyslipidemia, and level of kidney function were not associated with the cardiovascular outcome in this cohort (Table 2).

**Table 2:** Univariate cox-regression analysis for two-year cardiovascular outcome of 178 patients presented with chest pain to the emergency department

**Variable**	**Beta**	**SE**	**P-value**	**HR**	**95% CI**
Age (2 groups)	0.86	0.32	0.007	2.37	1.26-4.46
Hypertension (yes/no)	0.79	3.33	0.018	2.22	1.14-4.29
Prev. cv disease (yes/no)	1.33	3.37	<0.001	3.78	1.82-7.85
DM (yes/no)	-0.48	0.44	0.27	0.62	0.26-1.46
Dyslipidemia (yes/no)	-0.25	0.32	0.43	0.78	0.42-1.45.
hs-CRP (2 groups)	0.65	0.30	0.034	1.91	1.05-3.48
Plasma TNT (2 groups)	0.7	0.32	0.026	2.02	1.09-3.76
S. creatinine (2 groups)	0.24	0.3	0.43	1.27	0.71-2.29
Albuminuria (2 groups)	0.99	0.30	0.001	2.68	1.48-4.85
IgM-uria (2 groups)	1.08	0.34	0.001	2.96	1.52-5.77

**Beta:** Regression coefficient, **SE:** Standrad error of regression**, HR:** Hazard ratio**, 95% CI:** 95% confidence interval**, DM:** Diabetes mellitus, **IgM-uria:** Urine IgM creatinine ratio, hs-**CRP:** High sensitive C-reactive protein, **TNT:** Troponin T. **Prev. cv disease:** Previous cardiovascular disease.

In a multivariate Cox-regression analysis, a significant association remained between IgM-uria and the cardiovascular outcome even after adjustment for key potential confounding variables: age, hypertension, previous cardiovascular event, kidney function, and albuminuria, (HR = 2.1, 95% CI 1.05–4.01, p = 0.04).

**Figure 1:**
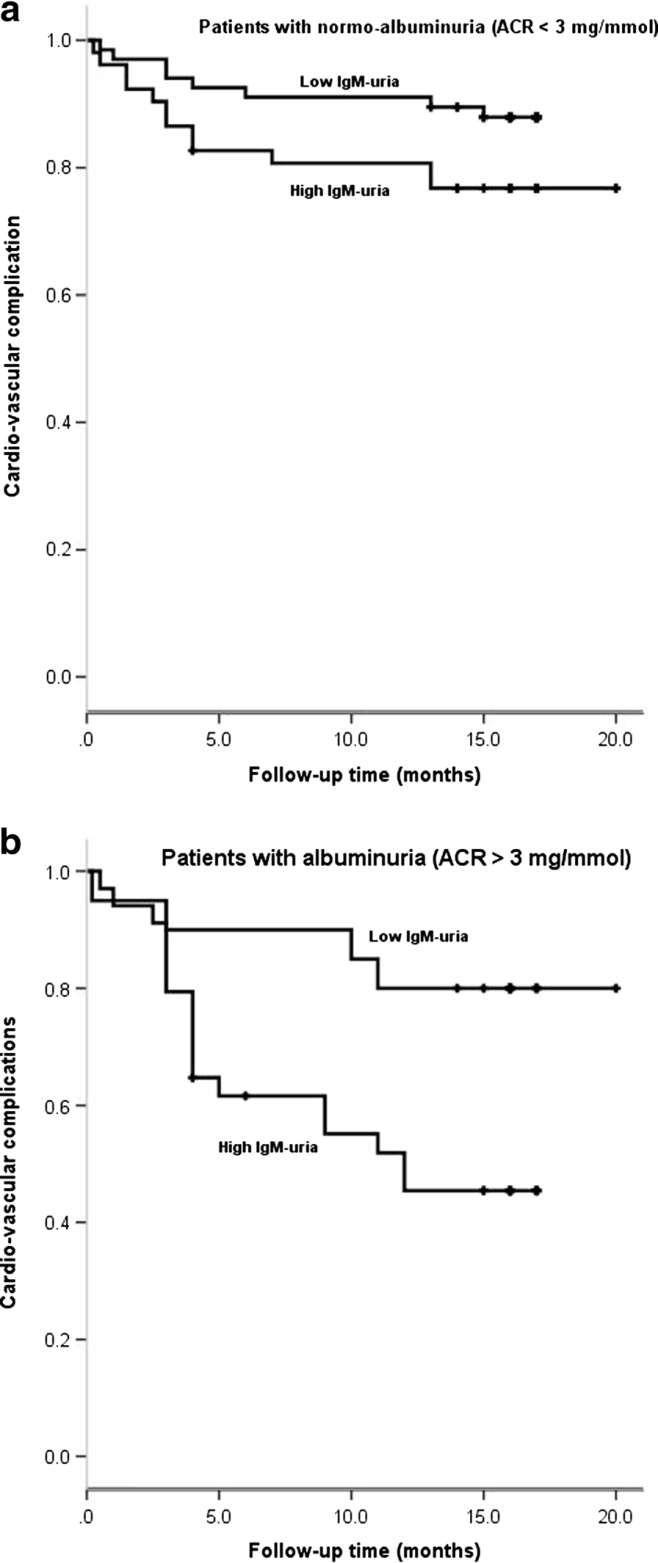
**Two-year cardiovascular outcome of 178 patients presented with chest pain stratified by the degree of IgM uria. A**: patients with normo-albuminuria (ACR < 3 mg/mmol). **B**: patients with albuminuria (ACR > 3 mg/mmol).

In Kaplan Meier curve analysis, patients with MA (≥3 mg/mmol) and high levels of IgM-uria (≥0.0054 mg/mmol) had a three-fold higher risk of new cardiovascular events than patients with MA and low-grade IgM-uria (RR = 3.3, 95% CI: 1.1-9.9, p *=* 0.001) (Figure 1).

## Discussion

In this prospective study, we found that patients who presented to the ED with chest pain due to acute coronary artery disease had a higher level of MA and IgM-uria than those with non-specific chest pain. Indeed, IgM-uria was associated with a higher risk of occurrence of subsequent major cardiovascular events. Furthermore, IgM-uria predicted poor long term cardiovascular outcome, independently of levels of MA, kidney function, diabetes and hypertension.

Under normal conditions the glomerular filtration barrier (GFB) allows just tiny amounts of albumin (molecular radius 35.5 Å) to pass to the primary urine, just one molecule out of 10,000 [18]. More than 97% of the filtered molecules are normally subsequently reabsorbed by the proximal tubules [19,20]. However, the GFB is not a static filter, but rather a highly dynamic sieve, which can directly increase its permeability in systemic inflammation [21], after trauma [22], during hyperglycemia [23], or following elevations of circulating ANP (atrial natriuretic peptide) levels [24], or angiotensin II levels [25]. Our data strongly suggest that albumin and other large proteins, such as IgG (mol. radius 55 Å) and IgM (mol. radius 120 Å) are able to pass the GFB only through large size-selective defects, “large pores” (shunts). An increased number of such shunt-like pores will functionally form in all of the pathophysiological conditions listed above resulting in albuminuria, IgG-uria and IgM-uria, while, at least initially, urinary tubular markers will stay unchanged. Consequently, the increased urinary IgM concentrations seen in patients with CAD must be secondary to decreases in size-selectivity of the GFB. Such size-selective defects are likely to be caused by endothelial dysfunction (due to atherosclerosis), acute systemic inflammation and/or ischemic (or HF-induced) release of ANP [20,24,26]. This is further strengthened by the fact that elevations of hs-CRP, reflecting systemic inflammation associated with atherosclerosis [27], and troponin T, more reflecting cardiac muscle ischemia, were coupled to the presence of CAD [28,29] and to MA and IgM-uria, in turn signaling a poor cardiovascular outcome [12,13]. Unfortunately, it is likely that the method for assessing IgG urine concentration may not have been sensitive enough compared with the methods for MA and IgM-uria, since IgG-uria was not as strongly coupled to predictions of outcome as were MA and IgM-uria. Urine IgM analysis is a simple, non-invasive urine test that could be incorporated in the routine laboratory analysis, for risk stratification of patients with chest pain [5,30]. Addition of urine IgM analysis to the workup of patients with coronary artery disease could help to identify patients at higher risk of recurrent cardiovascular events. This prompts early introduction of an intensive multifactorial preventive treatment strategy to improve the overall cardiovascular risk profile [31].

The study is limited by its observational nature. It was conducted in one centre and the participants were almost only of Scandinavian origin. However, our study has more than a few strengths. First, the patients were included prospectively and consecutively. Second, the study has a relatively long follow up time. Third, there was no loss of subjects during the follow up. Furthermore, urine IgM was measured during the first doctor contact in the ED.

## Conclusion

In conclusion, patients with chest pain, with MA and high levels of IgM-uria carry a higher probability for coronary artery disease and a poorer long-term cardiovascular outcome than patients with low IgM-uria. A larger longitudinal confirmatory study is needed to evaluate the usefulness of urine IgM in risk stratification of patients with Coronary Artery Disease.

## Competing interests

The authors declare that they have no competing interests.

## Authors’ contributions

RT and OB designed the study, analyzed the data, and draft the manuscript. UE, OT, PS, BR, contributed to the design of the study and drafting the manuscript. All authors read and approved the final manuscript.

## Pre-publication history

The pre-publication history for this paper can be accessed here:

http://www.biomedcentral.com/1471-2261/13/72/prepub
